# PATIENT PORTAL USE, PATIENT TRUST, SOCIAL DETERMINANTS OF HEALTH, AND SHARED DECISION-MAKING AMONG US OLDER ADULTS

**DOI:** 10.1093/geroni/igae098.2637

**Published:** 2024-12-31

**Authors:** Yuling Chen

**Affiliations:** Johns Hopkins University, Baltimore, Maryland, United States

## Abstract

**Background:**

Shared decision-making (SDM) and patient portals are increasingly embraced in health care and recommended in cardiovascular disease (CVD) guidelines. This study investigated the associations between patient portal use, patient trust in the healthcare system, social determinants of health (SDOH), and patient involvement in SDM among adults 65 years or older with or at risk of CVD.

**Methods:**

We conducted a cross-sectional study of US adults from the 2022 Health Information National Trends Survey. CVD risk was defined as reporting a heart condition, diabetes, hypertension, and/or current smoking. SDM was measured by asking how often patients’ providers involved them in health decisions over the past year. A weighted multivariable logistic regression model was employed, adjusting for sociodemographic and clinical characteristics.

**Results:**

The study included 1,139 older adults with or at risk of CVD, with a mean age of 73.5 (SD 6.7) years. The sample included 54.0% females; 66.4% identified as white. Older adults who accessed the patient portal 6 to 9 times in the past year were 1.98 times more likely to engage in SDM (95% CI 1.13-3.48) compared to those without access. Older adults with high trust in the health system were 3.15 times more likely to engage in SDM (95% CI 1.89-5.23) than those with low trust. However, SDOH (inability to afford meals, being forced to move, and lack of transportation) was not significantly associated with SDM.

**Conclusion:**

These findings underscore the vital role of utilizing patient portals and fostering trust in enhancing older adults’ engagement in SDM.

